# Incidence and trends of limb amputation in first nations and general population in Saskatchewan, 2006–2019

**DOI:** 10.1371/journal.pone.0254543

**Published:** 2021-07-12

**Authors:** Samuel Kwaku Essien, Gary Linassi, Margaret Larocque, Audrey Zucker-Levin

**Affiliations:** 1 School of Rehabilitation Science, University of Saskatchewan, Saskatoon, SK, Canada; 2 Department of Physical Medicine and Rehabilitation, University of Saskatchewan, Saskatoon, SK, Canada; 3 Patient-Oriented Research Team (PORT), Saskatoon, SK, Canada; McGill University, CANADA

## Abstract

**Background:**

There is conflicting evidence whether limb amputation (LA) disproportionately affects indigenous populations. To better understand this disparity, we compared the LA incidence rate between First Nations persons registered under the Indian Act of Canada (RI) and the general population (GP) in Saskatchewan.

**Methods:**

We used Saskatchewan’s retrospective administrative data containing hospital discharge LA cases, demographic characteristics (age and sex), and residents population reported in the database stratified by RI and GP from 2006–2019. The LA cases for each stratified group were first disaggregated into three broad categories: overall LA (all reported LA), primary LA (first reported LA), and subsequent LA (revision or contralateral LA), with each category further split into the level of amputation defined as major amputation (through/above the ankle/wrist joint) and minor amputation (below the ankle/wrist joint). LA rates were calculated using LA cases as the numerator and resident population as the denominator. Joinpoint and negative binomial regressions were performed to explore the trends further.

**Results:**

Overall, there were 1347 RI and 4520 GP LA cases reported in Saskatchewan from 2006–2019. Primary LA made up approximately 64.5% (869) of RI and 74.5% (3369) of GP cases, while subsequent LA constituted 35.5% (478) of RI and 25.5% (1151) of GP cases. The average age-adjusted LA rate was 153.9 ± 17.3 per 100,000 in the RI cohort and 31.1 ± 2.3 per 100,000 in the GP cohort. Overall and primary LA rates for the GP Group declined 0.7% and 1.0%, while subsequent LA increased 0.1%. An increased LA rate for all categories (overall 4.9%, primary 5.1%, and subsequent 4.6%) was identified in the RI group. Overall, minor and major LA increased by 6.2% and 3.3%, respectively, in the RI group compared to a 0.8% rise in minor LA and a 6.3% decline in major LA in the GP group. RI females and males were 1.98–1.66 times higher risk of LA than their GP counterparts likewise, RI aged 0–49 years and 50+ years were 2.04–5.33 times higher risk of LA than their GP cohort. Diabetes mellitus (DM) was the most prevalent amputation predisposing factor in both groups with 81.5% of RI and 54.1% of GP diagnosed with DM. Also, the highest proportion of LA was found in the lowest income quintile for both groups (68.7% for RI and 45.3% for GP).

**Conclusion:**

Saskatchewan’s indigenous individuals, specifically First Nations persons registered under the Indian Act of Canada, experience LA at a higher rate than the general population. This disparity exists for all variables examined, including overall, primary, and subsequent LA rates, level of amputation, sex, and age. Amplification of the disparities will continue if the rates of change maintain their current trajectories. These results underscore the need for a better understanding of underlying causes to develop a targeted intervention in these groups.

## Introduction

Limb amputation (LA), the surgical removal of part of or an entire limb, is usually performed to prevent deterioration of an individual’s health [1, 2]. Unfortunately, LA does not solely affect individuals physically but has a multifaceted impact on quality of life, including significant social, psychological, cultural, and economic burdens placed on the patient, family, and caregivers [3–5].

The multidisciplinary patient-oriented research team (PORT), comprised of people with amputation, caregivers, researchers, educators, and health care providers was created to focus on amputee health and well-being in Saskatchewan, Canada. Among the highest priorities, the PORT identified the need to understand the specific epidemiology of LA as it relates to Indigenous communities in Saskatchewan with the ultimate goal to determine how Saskatchewan LA rates compare to other Canadian provinces and globally [6].

Zhang recently reported an estimated 1.8% of the total global population are living with a lower-extremity disability, including amputation with a rising prevalence of LA in both resource-limited and resource-rich countries, of which Canada is not an exception [7–10]. The upsurge in the prevalence of LA observed in these countries is predominantly caused by peripheral arterial disease (PAD) in the presence of diabetes mellitus (DM) [9–11].

Saskatchewan has a population of approximately 1.1 million people, of which 15% identify as Indigenous (10% First Nations, 5% Métis and 0.2% Inuit), the second-highest per capita Indigenous population among Canadian provinces [12]. Diabetes Canada reports approximately 8.0% of the Saskatchewan population are diagnosed with DM, with twice as many diagnosed with pre-diabetes [13]. This is of particular concern, as the number of Saskatchewanians diagnosed with DM is expected to increase to 10% over the next decade [13].

There is evidence that indigenous populations, including First Nations, suffer from DM at a greater rate than non-indigenous populations, but there is conflicting evidence whether LA disproportionately affects indigenous populations [14–16]. Norman et al. (2010) found a higher rate of diabetes-related minor LA in Western Australia’s Indigenous people when compared to Non-Indigenous people [16] In contrast, Gurney et al. found no increased risk of diabetes-related minor LA in New Zealand’s Māori Indigenous people [15].

These conflicting reports lead to the question of whether DM has a differential impact on LA in Saskatchewan. No known research has explicitly compared the epidemiology and current trends of LA among First Nations and the general population in Saskatchewan, with the overarching goal of identifying the challenges and needs of Saskatchewan amputees.

This study aimed to compare the LA incidence rate between First Nations persons registered under the Indian Act of Canada [17] and the general population in Saskatchewan.

## Methods

### Data

Retrospective incidence of amputations was examined using linked administrative health data from the Province of Saskatchewan, Canada, between January 1, 2006, to December 31, 2019. Saskatchewan has a universal single-payer health care system and administrative health databases that documents health services utilization. We accessed two datasets to conduct this study: Person Health Registration System (PHRS)–sociodemographic and health coverage information and Discharge Abstracts Database (DAD)–acute-care hospital information. These databases were accessed through the Saskatchewan Health Quality Council and were electronically linked using encrypted, unique identification numbers.

Demographic characteristics and amputation procedure codes listed in the Canadian Classification for Health Intervention codes were used (1SN93, 1SQ93, 1TA93, 1TK93, 1TM93, 1TV93, 1VA93, 1VC93, 1VG93, 1VQ93, 1UB93, 1UE93, 1UF93, 1UG93, 1UH93, 1UI93, 1UJ93, 1UK93, 1UM93, 1WA93, 1WE93, 1WI93, 1WJ93, 1WK93, 1WL93, 1WM93, 1WN93) [18]. The inclusion criterion was amputation code found in any of the 20 designated intervention fields. Data completeness, reliability, and validity have been reported elsewhere [19–21]. It is not possible to identify people’s ethnicity in administrative databases, but it is possible to identify First Nations persons registered under the Indian Act of Canada, further referred to as Registered Indians (RI) [17]. This legal identity variable is documented in the PHRS when individuals apply to have health care coverage, specifically when applying for their provincial health card. New Saskatchewan residents are required to register themselves and their dependents for a Saskatchewan Health Card to receive health benefits. Applicants are asked to declare if they have FN status and to provide the supporting document (i.e., certificate of Indian Status). Since 2010, individuals have the option to declare or not if they have FN status. The 2016 Canadian census identifies 106,440 people with RI status representing 60.8% of the 175,015 Indigenous residents of Saskatchewan [22]. All other Saskatchewan residents including whites, immigrants, Indigenous people who are non- Registered Indian or have chosen not to self-identify, Métis and Inuits will be identified in the General Population (GP) cohort of our study allowing us to compare amputation rates between Registered Indians and General Population.

Amputation cases were divided into three groups: (1) overall amputation cases (includes all LA cases), (2) primary amputation cases (the first report of LA in an individual), and (3) subsequent amputations (any report of an additional LA, revision or contralateral, in an individual identified in the primary amputation group). These groups were further subdivided into major amputation (through/above the ankle/wrist joint) and minor amputation (below the ankle/wrist joint) [23]. Amputation cases for each population group (RI and GP) were further stratified by sex (female/male), and age (0–49 years and 50+ years). The direct cause of LA could not be identified from the dataset thus Amputation Predisposing Factors (APF) based on co-morbidities present at the time of LA were used as proxy. ICD-10-CA diagnostic codes including diabetes (e.g., E10-E14), vascular diseases (e.g., I70, I72-I78, and I80-I99) and trauma (e.g., S480-S481, S680-S684, S980-S984, and T050-T059) were selected for assessment as these are among the most common APF. Additional APF including infection, cancer and congenital were considered in the “other” category as small cell size precluded further analysis. The Saskatchewan residents population stratified by RI and GP reported in the administrative database was used as denominators for the rate calculations. This study received ethical approval from the University of Saskatchewan Biomedical Ethics Board (U of S # Bio1590).

### Data analysis

To calculate the yearly LA rates and compare between the two populations, LA cases by year for each population group were divided by each group’s annual Saskatchewan resident population. Age-and-sex adjusted rates were standardized to the 2011 Saskatchewan population via the direct standardization method [24]. The standardization procedure was carried out by multiplying age and sex-groups LA specific rates by the specific weights of age and sex in the 2011 Saskatchewan population [25]. T-tests were performed to determine if LA rates differed between RI and GP overall, primary, and subsequent LA. As well as major and minor LA with significance set at p < 0.05.

Also, a Joinpoint regression [26] was used to test and identify significant changes in LA rate trends over the eighteen years investigated (2006–2019). The grid search method and the permutation test built-in the Joinpoint software were used, respectively, to identify the best model fit and the optimal number of breakpoints [27]. The 95% confidence interval (Cl), annual percent change (APC), and the average annual percent change (AAPC) were determined from each model fit.

Finally, a negative binomial regression [28] was used to test for significant differences in LA rate trends over time between female/male, age 0-49/50+ years, minor and major LA. A bivariate model was first fitted between rates of amputation and each demographic factors (e.g., age and sex). After that, a separate multivariate model with an interaction term created by multiplying amputation year by each factor was performed. This model approach has successfully been used in the United States to compare regional differences in anaphylaxis hospitalization rate [29]. The 95% confidence interval (CI) and the relative rate (RR) were estimated from the models. A statistically significant difference in LA rates trends was determined by a p-value of interaction term <0.05. Confounding was assessed using the change in coefficient estimate approach, where more than 10% change between the adjusted and unadjusted estimates was deemed a confounder [30].

## Results

From 2006 to 2019, there were 1347 RI and 4520 GP LA cases reported in Saskatchewan. Of these numbers, approximately 64.5% (869) of RI LA cases and 74.5% (3369) of GP LA cases were primary amputation cases while subsequent amputation constituted 35.5% (478) of RI and 25.5% (1151) of GP cases. Approximately, 64% of RI and 80% of GP were aged 50+ years. Males constituted the largest share of both RI (68%) and GP (72%) cohorts. As shown in Table 1, DM was the highest APF reported in both groups with 81.5% of RI and 54.1% of GP diagnosed with DM. More people in the GP (16.3%) than in the RI (4.2%) group had a history of vascular disease independent of DM and trauma was higher in the GP (8.7%) than the RI (4.3%) group. Further, both groups reported the highest number of LA in the lowest income quintile group (68.7% for RI and 45.3% for GP).

**Table 1:** Characteristics of amputation predisposing factors and income quintiles.

Variables	RI	GP
	N (%)	N (%)
**Predisposing Factors**		
Diabetes	1098 (81.5%)	2447 (54.1%)
Peripheral vascular disease (PVD)	56 (4.2%)	734 (16.3%)
Trauma	58 (4.3%)	393 (8.7%)
Other	135 (10.0%)	946 (20.9%)
**Income Quantiles**		
Highest	101 (7.5%)	620 (13.7%)
Middle	236 (17.5%)	1605 (35.5%)
Lowest	925 (68.7%)	2049 (45.3%)
Not reported	85 (6.3%)	246 (5.5%)

**Fig 1:**
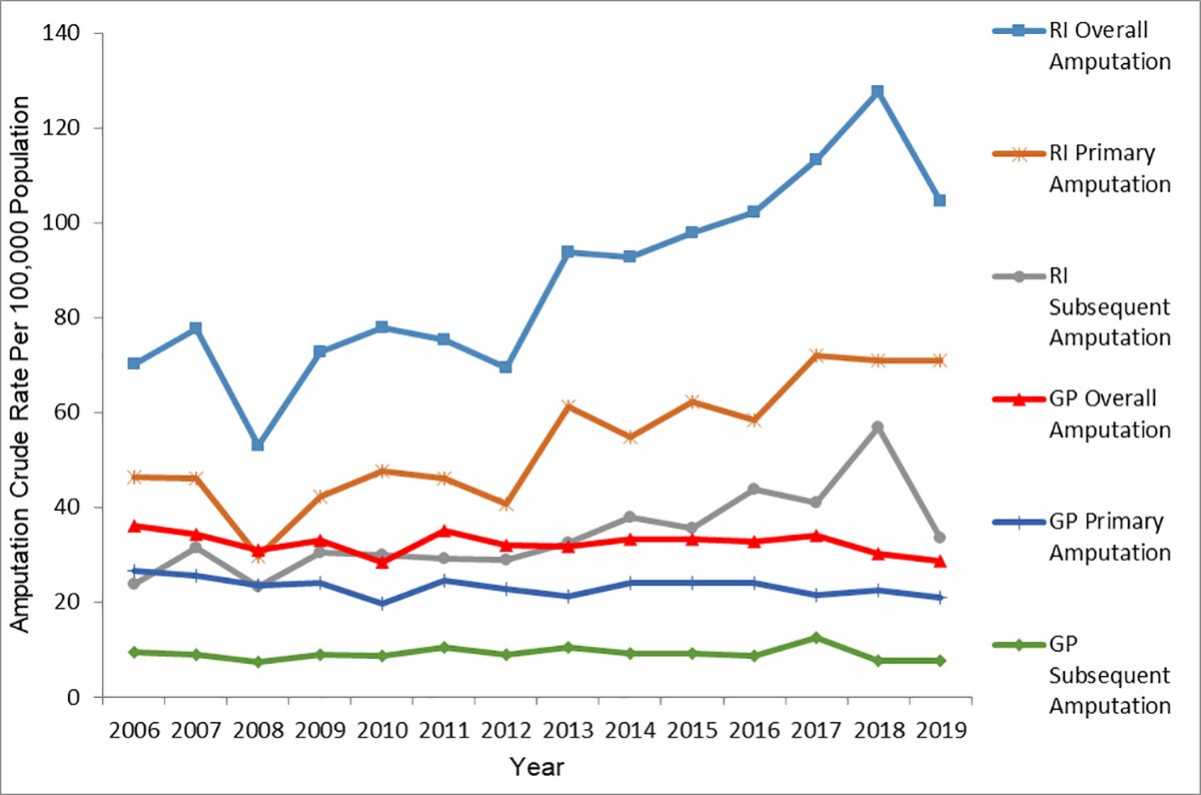
RI and GP overall, primary, and subsequent amputation crude rates 2006–2019.

Fig 1 demonstrates that during the study period, the average overall LA rate was 87.7 ± 20.2 per 100,000 population in the RI cohort and 32.4 ± 2.3 per 100,000 in the GP cohort. The average primary LA rate was 53.6 ± 12.9 per 100,000 population in the RI cohort and 23.3 ± 1.9 per 100,000 in the GP cohort. The subsequent LA rate was 34.2 ± 8.7 per 100,000 population in the RI cohort and 9.2 ± 1.4 per 100,000 in the GP cohort. T-tests revealed the overall, primary, and subsequent LA rates were all significantly higher in the RI cohort compared to the GP cohort (p<0.001).

During the interval (2006–2019), the highest LA rate in RI and GP occurred in 2018 (127.72 per 100,000 population) and 2006 (36.19 per 100,000 population), respectively. The lowest LA rates in RI and GP occurred in 2008 (53.11 per 100, 000 population) and 2010 (28.51 per 100, 000 population) respectively. The highest rate observed in RI in 2018 also coincided with the highest RI subsequent LA rate (56.87 per 100, 000 population). However, RI primary LA rate during the same time (2017–2019) remained stable. This suggesting that RI subsequent LA cases in 2018 contributed immensely to the highest overall RI rate observed in 2018. GP rates consistently remained stable from 2011 to 2017 before experiencing a sharp decline between 2017 and 2019. The 2017 to 2019 fall in GP rates reflects the decline observed in both primary and subsequent GP rates during the same time period.

Joinpoint analysis (Table 2) showed a 0.7% decline in the overall GP LA rates from 2006 to 2019 while the overall RI LA rate increased 4.9% during the same interval. The rate of primary GP LA decreased 1.0% over the study period while the RI Primary LA rate increased 5.1% during the same time interval. Subsequent LA for both RI and GP cohorts increased 4.6% and 0.1% respectively over the study period.

**Table 2:** Annual percent change in registered Indians and general populations amputation rates, 2006–2019.

Rate	Breakpoints	APC(95%Cl)	Full Range	
			AAPC	(95%Cl)
RI Overall Amputation	2006–2019	4.9*(3.1 to 6.8)	4.9*	(3.1 to 6.8)
GP Overall Amputation	2006–2019	-0.7 (-1.6 to 0.3)	-0.7	(-1.6 to 0.3)
RI Primary Amputation	2006–2019	5.1*(2.9 to 7.4)	5.1*	(2.9 to 7.4)
GP Primary Amputation	2006–2019	-1.0 (-2.1 to 0.0)	-1.0*	(-2.1 to 0.0)
RI Subsequent Amputation	2006–2019	4.6* (2.3 to 6.9)	4.6*	(2.3 to 6.9)
GP Subsequent Amputation	2006–2019	0.1 (-2.0 to 2.2)	0.1	(-2.0 to 2.2)
RI Overall Major Amputation	2006–2019	3.3* (0.5 to 6.3)	3.3*	(0.5 to 6.3)
GP Overall Major Amputation	2006–2010	-12.0* (-19.8 to -3.5)	-6.3*	(-10.6 to -1.7)
	2010–2017	3.2 (-1.8 to 8.5)		
	2017–2019	-24.0 (-43.4 to 2.0)		
RI Primary Major Amputation	2006–2008	-29.6 (-52.9 to 5.1)	0.5	(-5.0 to 6.4)
	2008–2019	7.3* (4.4 to 10.2)		
GP Primary Major Amputation	2006–2019	-3.6* (-6.1 to -1.0)	-3.6*	(-6.1 to -1.0)
RI Overall Minor Amputation	2006–2019	6.2* (3.9 to 8.5)	6.2*	(3.9 to 8.5)
GP Overall Minor Amputation	2006–2019	0.8 (0.0 to 1.7)	0.8	(0.0 to 1.7)
RI Primary Minor Amputation	2006–2019	6.1* (3.9 to 8.4)	6.1*	(3.9 to 8.4)
GP Primary Minor Amputation	2006–2019	0.3 (-0.5 to 1.1)	0.3	(-0.5 to 1.1)

APC-Annual Percent Change, AAPC- Average Annual Percent Change, Cl-Confidence Interval.

*Indicates a statistically significant breakpoint.

**Fig 2:**
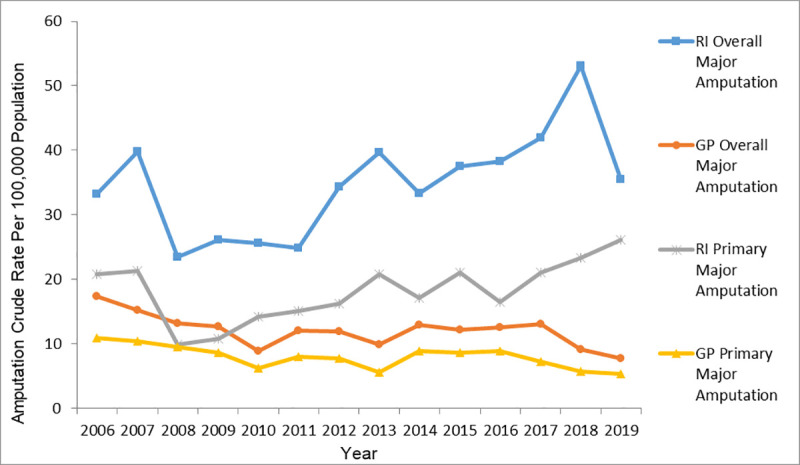
RI and GP overall and primary major amputation crude rates 2006–2019.

Fig 2 illustrates the trends in major LA rates from 2006 to 2019 in the RI and GP cohorts. The average overall major LA rate was 34.8 ± 8.1 per 100,000 in the RI cohort and 12.1 ± 2.5 per 100,000 in the GP cohort. The average primary major LA rate was 18.1 ± 4.7 per 100,000 in the RI cohort and 8.0 ± 1.8 per 100,000 in the GP cohort. T-tests revealed the overall and primary major LA rates were significantly higher in the RI cohort compared to the GP cohort (p<0.001).

Joinpoint analysis revealed contrasting trends in overall major LA rate between the GP and RI cohorts (Table 2). The AAPC shows the rate of GP overall major LA and GP primary major LA decreased 6.3% and 3.6% respectively between 2006 and 2019. This is in contrast to that of the RI cohort with significant increase 3.3% identified for RI overall major LA and no significant change for RI primary major LA during the same time interval. Breakpoints were identified within study period for RI primary major LA revealing an insignificant 29.6% decrease from 2006–2008 countered by a significant 7.3% increase for the time period 2008–2019. Due to limited samples, subsequent major and minor LA’s were not further explored.

**Fig 3:**
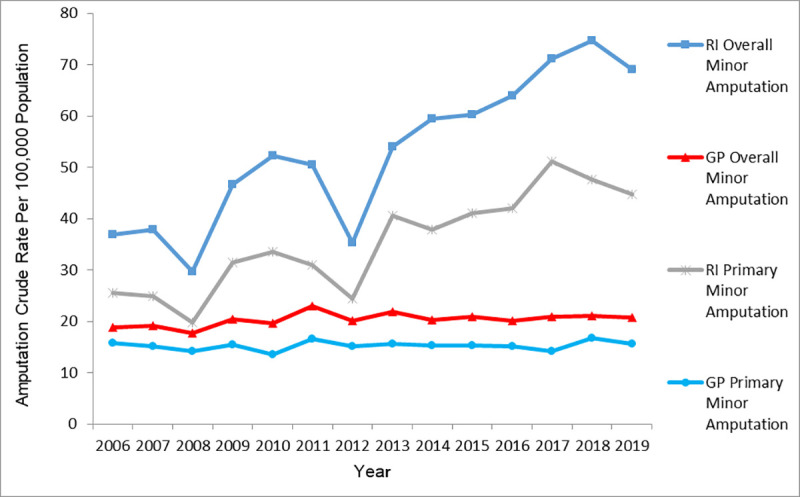
RI and GP overall and primary minor amputation crude rates 2006–2019.

Fig 3 illustrates the trends in minor LA rates from 2006 to 2019 in the RI and GP cohorts. The average overall minor LA rate was 52.9 ± 14.3 per 100,000 in the RI cohort and 20.3 ± 1.3 per 100,000 in the GP cohort. The average primary minor LA rate was 35.4 ± 9.6 per 100,000 in the RI cohort and 15.3 ± 0.9 per 100,000 in the GP cohort. T-tests revealed the overall minor and primary minor LA rates were significantly higher in the RI cohort compared to the GP cohort (p<0.001).

Joinpoint regression identified significant rising rates of overall minor LA (6.2%) and primary minor LA (6.1%) in the RI cohort but 0.8% and 0.3% no signiicant rise in the GP overall primary minor LA rate during the 2006–2019 study period.

### Age and sex crude and adjusted rates

**Fig 4:**
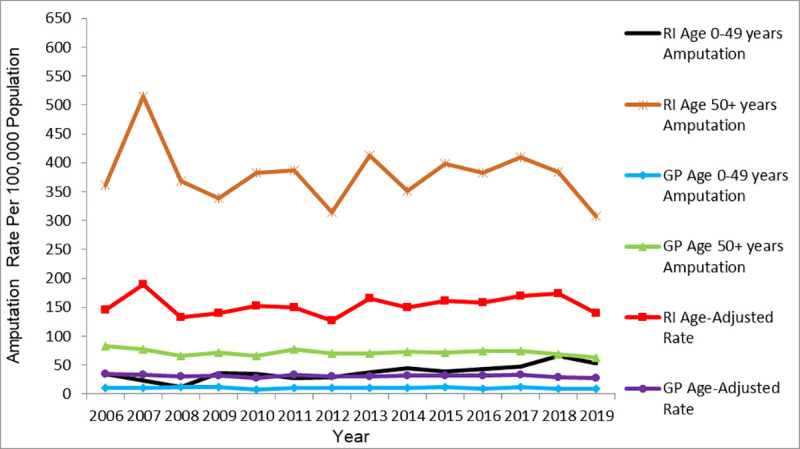
RI and GP age-adjusted and age-stratified crude amputation rates by year.

**Fig 5:**
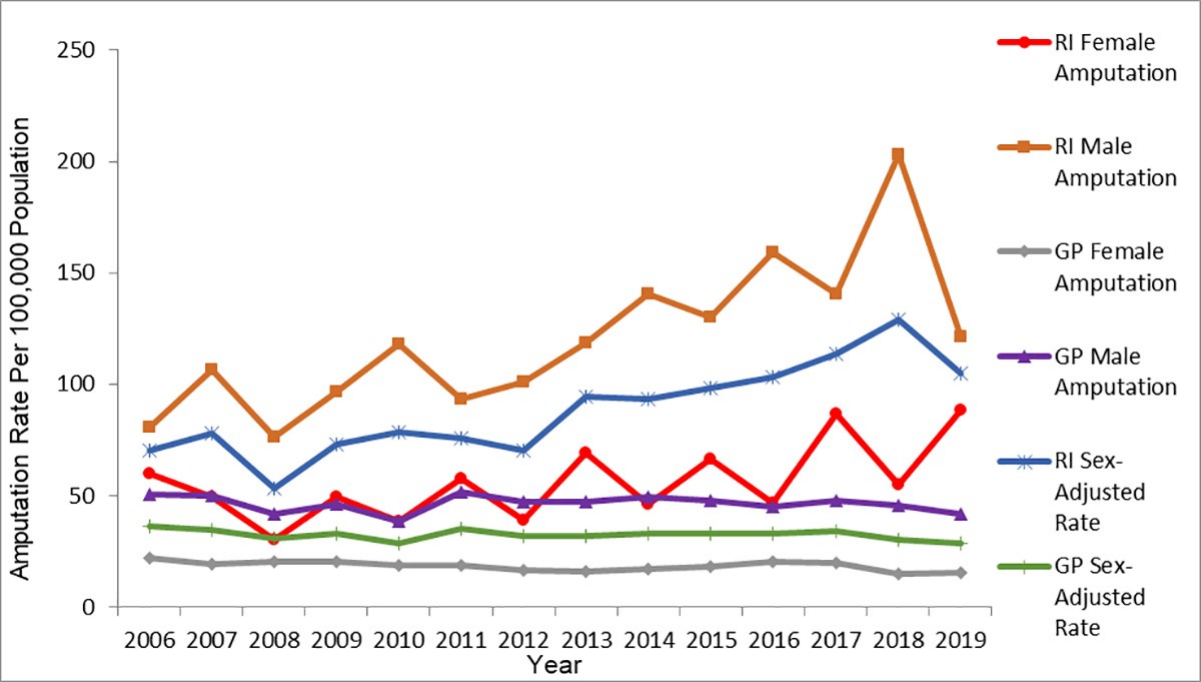
RI and GP sex-adjusted and sex-stratified crude amputation rates by year.

Figs 4 and 5 present the RI and GP age- and sex-stratified crude LA rates and adjusted overall LA rates from 2006 to 2019. The calculated average age-adjusted LA rate from Fig 4 higher adjusted rates of LA in the RI cohort (153.9 ± 17.3 per 100,000) than in the GP cohort (31.1 ± 2.3 per 100,000). The RI age-adjusted rate showed an increasing trend in rate between 2006 and 2007 and a declining trend after 2007. In contrast, GP age-adjusted rate remained stable over the study years. In both RI and GP, higher crude rates of LA were seen in those aged 50+ years than in those aged 0–49 years.

Fig 5 revealed higher crude rates of LA in males than females in both the RI and GP cohorts. The calculated average sex-adjusted LA rate also revealed higher adjusted rates of LA in the RI cohort (88.2 ± 20.5 per 100,000 population) than in the GP cohort (32.4 ± 2.3 per 100, 000 population).

Table 3 summarizes the unadjusted and adjusted relative rates comparing amputation incidences among RI and GP populations. The adjusted *model I* results show that among individuals 0–49 years of age, RI were twice as likely as GP to have LA (adjusted RR = 2.04, 95%CI 1.36–3.08) and among individuals 50+ years of age, RI were 5.33 times as likely as GP to have LA (adjusted RR = 5.33, 95% CI 4.38–6.50, p<0.001). The overall crude age rates of change over time did not differ among RI and GP individuals aged 50+ years, as identified by the non-significant age- and year of LA interaction (p = 0.924). However, the overall crude age rates of change over time did differ among RI and GP individuals aged 0–49 years (p = 0.002).

**Table 3:** Unadjusted and adjusted relative rates comparing amputation incidences among registered Indians and general populations.

**Variables**	**Model I (AGE Comparison)**							
	**Unadjusted**							
**Status**	**0–49 years (R**^**2**^ **= 0.29)**				**50+ years (R**^**2**^ **= 0.25)**			
	**Coef.**	**RR**	**95%Cl**	**P-value**	**Coef.**	**RR**	**95%Cl**	**P-value**
GP (ref)		1.00		<0.001		1.00		<0.001
RI	1.312	3.72	(2.88–4.79)		1.665	5.29	(4.80–5.82)	
	**Adjusted**							
GP (ref)		1.00				1.00		<0.001
RI	0.715	2.04	(1.36–3.08)	0.001	1.674	5.33	(4.38–6.50)	
**Year**	-0.083			0.061	-0.006			0.780
Status and Year Int*	0.075			0.002	-0.001			0.924
**Model II (SEX Comparison)**								
**Status**	**Unadjusted**							
	**Female (R**^**2**^ **= 0.24)**				**Male (R**^**2**^ **= 0.25)**			
GP (ref)		1.00		<0.001		1.00		<0.001
RI	1.120	3.06	(2.51–3.75)		0.951	2.59	(2.21–3.04)	
	**Adjusted**							
GP (ref)		1.00		<0.001		1.00		<0.001
RI	0.682	1.98	(1.36–2.87)		0.509	1.66	(1.30–2.13)	
**Year**	-0.074			0.056	-0.059			0.016
Sex and Year Int*	0.057			0.011	0.056			<0.001
**Model III (AMPUTATION LEVEL Comparison)**								
**Status**	**Unadjusted**							
	**Major (R**^**2**^ **= 0.26)**				**Minor (R**^**2**^ **= 0.30)**			
GP (ref)		1.00				1.00		<0.001
RI	1.057	2.88	(2.38–3.49)	<0.001	0.958	2.61	(2.17–3.12)	
	**Adjusted**							
GP (ref)		1.00				1.00		<0.001
RI	0.569	1.77	(1.23–2.54)	0.002	0.549	1.73	(1.28–2.34)	
**Year**	-0.097			0.015	-0.043			0.165
Amputation level and Year Int*	0.065			0.004	0.051			0.003

RR-Relative Rate, Cl- Confidence Interval, Int* -Interaction, Coef-Coefficient

The adjusted *model II* results show that RI females were almost twice as likely than GP females to have LA (adjusted RR = 1.98, 95%CI 1.36–2.87) and RI males were at a higher risk of LA compared to GP males (adjusted RR = 1.66, 95% CI 1.30–2.13) (p = <0.001). Further, the overall crude male and female LA rates of change over time did not differ for both RI and GP males (p = 0.011) and females (p<0.001).

Adjusted *model III* shows RI were more likely than GP to have major LA (adjusted RR = 1.77, 95% CI 1.23–2.54, p = 0.002) and minor LA (adjusted RR = 1.73, 95% CI 1.28–2.34, p<0.001). Further, the overall crude major and minor LA rates of change over time investigated did not differ for both RI and GP major (p = 0.004) and minor LA (p = 0.003). The further analysis presented in Table 4 identifies RI’s diagnosed with DM were 1.7 times more likely to have LA than GP’s diagnosed with DM.

**Table 4:** Unadjusted and adjusted relative rates comparing amputation incidences diagnosed with diabetes at the time of amputation among registered Indians and general populations.

DIAGNOSED WITH DIABETES AT THE TIME OF AMPUTATION								
	Unadjusted				Adjusted			
	Coef.	RR	95%CI	P-value	Coef.	RR	95%Cl	P-value
**Status**								
GP (ref)		1.00				1.00		
RI	0.443	1.56	(1.41–1.72)	<0.001	0.538	1.71	(1.38–2.12)	<0.001
**Year**	0.015			0.205	-0.002			0.895
Status and Year Int*					0.012			0.323

RR-Relative Rate, Cl- Confidence Interval, Int* -Interaction, Coef-Coefficient

The assessment of confounding based on the change in coefficient estimate approach yielded a magnitude of confounding of 21.5%. This indicated that the adjusted coefficient estimate for RI versus GP with a diagnosis of DM at the time of LA changed more than 10% from the unadjusted coefficient, hence, identifying DM as a confounder. The direction of the magnitude of this confounding value was negative indicating an underestimation of the true association between LA and DM diagnosis.

## Discussion

In this study, we analyzed Saskatchewan administrative health data and found striking disparities in LA rates which were 1.7–4.2 times higher in RI compared to GP Saskatchewanians from 2006–2019. Specifically, the average LA rate was significantly higher for overall, primary and subsequent LA in the RI cohort (87.7 ± 20.2, 53.6 ± 12.9, 34.2 ± 8.7 per 100,000 respectively) compared to GP cohort (32.4 ± 2.3, 23.3 ± 1.9, 9.2 ± 1.4 per 100,000 respectively). Further, AAPC increased 4.9% for overall LA during the study period in the RI cohort which drastically contrasted with the 0.7% decrease found in the GP cohort. This trend was largely driven by increases in primary LA (5.1%) in the RI cohort and decrease (1.0%) in the GP cohort. Although both cohorts had an AAPC increase in subsequent LA rates (RI 4.6%, GP: 0.1%) during the study period, of particular concern is the rise in primary LA rates from 2013–2019 in the RI cohort. Further analysis of overall and primary major and minor LA rates were not examined due to limited samples. This analysis identified a decrease rate of 6.3% for overall major LA in the GP cohort largely driven by a 3.6% decrease in primary major LA while the rate of overall minor LA increased 0.8% with no change in primary minor LA rate. In contrast, even though overall major LA rate increased by 3.3% in the RI cohort during the study period, no significant change in rate was identified for primary major LA during the same time interval. This suggests that the increase in overall major LA was not driven by primary major LA but possibly by subsequent major LA. The increase in AAPC by 6.2% in RI overall minor LA was supported by the rise in primary minor LA during the study years investigated. These data indicate that the increase in overall LA in the RI cohort is due to more minor LA’s performed whereas the decrease in overall LA in the GP cohort is due to fewer major LA’s performed over the study period.

The difference in LA rate may be explained by difference in comorbidity between the two populations. Diabetes, the leading cause of amputation, affects Indigenous populations at a higher rate than GP populations in Ontario, Alberta, Manitoba, and Saskatchewan [14, 31–34].

Our finding that the rate of LA among Saskatchewan RI is 1.7 times higher than GP are supported by observations of LA in RI populations related to diabetes in other Canadian provinces [35–37]. Shah et al. (2019) examined Ontarians with diabetes and found a 3–5 times higher risk of lower extremity amputation (LEA) in RI than GP populations [35]. Similarly, a study in Alberta found a 3 times higher average age-adjusted rate of LEA in First Nations than Non-First Nations populations with diabetes [36]. Finally, a published report identified diabetes complication-related LA in Manitoba First Nation individuals are 18 times higher than other Manitoban populations [37]. Although the present study did not specifically focus on LEA or diabetes, the reported disparities in LA rates between RI and GP populations support our findings even though the data is incomparable in the sense that population proportions were not adjusted for. Elsewhere, Rodrigues et al. (2016) found higher proportions of LEA in Indigenous Australians (56.5%) than Non-Indigenous Australians (29.2%) [38].

Our finding that males, both RI and GP, had a higher incidence of LA than females throughout the study period is well supported in the literature [15, 36], however, O’Rourke et al. (2013) instead found more cases of LA in Indigenous females than males [39]. Our findings may be attributed to more frequent foot self-care which is essential to mitigate ulcerations performed by women with diabetes than men [40] and that women are more timely and higher users of primary care, including diabetic foot care [15, 41].

Notwithstanding the difference in the overall LA rates between the two populations (RI and GP), individuals aged 50+ years in both populations had higher rates of LA than those aged 0–49 years. These findings are consistent with those reported in Alberta with LA cases increasing with age, and 47% of LA cases in both RI and GP populations occurred in those aged 65–84 years [35]. Within the age groups, RI were more likely to have LA than GP with RI individuals 0–49 years of age 2.04 times more likely as GP to have LA and RI individuals 50+ years of age 5.33 times more likely as GP to have LA.

The higher RI overall LA rates observed between 2006 and 2019 were supported by a substantially higher average overall major and minor LA rates among RI people than for GP people. This observation is in line with the findings of O’Rourke who reported more frequent major LA in the diabetic Indigenous population of Northern Queensland, Australia [39]. Further, Norman et al. (2010) found a higher rate of both diabetic minor and major LA in Indigenous when compared to Non-Indigenous residents of Western Australia [16]. Likewise, Gurney reported higher rates of major LA (6.4 cases/1,000) and minor LA (7.1 cases/1,000) in New Zealand’s Māori Indigenous people when compared to Non-Indigenous groups [15].

Finally, the overall distribution of income and LA identified a similar negative trend between the RI and GP groups with the proportion of LA increasing in lower income quantiles. However, we found the proportion of LA differed between the groups with a higher percentage of LA in the lower income quantile in the RI than the GP group (68.7% versus 45.3% respectively). Data on Educational level was not available within our database but the 2016 Statcan report identifies income as proportional to education among Saskatchewanians who identify as aboriginal [42]. Further support for the disparity between income, education and access to health care are reported by Nguyen (2020) [43] who identified health care barriers faced by Indigenous Canadians that may have contributed to our findings of higher rates of major and minor LA in all groups (overall, primary and subsequent LA) in younger RI’s living in Saskatchewan.

## Strengths and limitations

This study presents with important strengths. To the best of the authors’ knowledge, this is the first provincial wide study to compare trends of LA between RI and GP populations. This study also adds to studies on LA in RI and GP in other Canadian provinces by broadly considering all types of amputations from all-causes but not limited to diabetes. The study also had some limitations. Due to limited samples, subsequent major and minor LA’s were not further explored in this study. Even though the proportions of patients with trauma, PVD and income quintiles were accounted for in this study, however, small cell size especially in the RI group precluded the comparison of LA in RI and GP rates stratified by trauma, PVD and income quintiles in the fourteen years and as well specific sites of amputation (e.g., trans-phalangeal, trans-tarsal, trans-tibial).

## Conclusion

Indigenous individuals, specifically First Nations persons registered under the Indian Act of Canada experience LA at a higher rate than the general population. This disparity exist in all aspects examined including overall, primary and subsequent LA rates, level of amputation, sex, and age. This is even more significant as First Nations persons registered under the Indian Act of Canada only represent 61% of the Aboriginal population of Saskatchewan leaving 39% of the Aboriginal population including First Nations persons not registered under the Indian Act, Métis and Inuit people, in the general population cohort.

The finding that the overall LA rates are decreasing, largely due to a decline in major LA rates in GP Saskatchewanians is positive, however it contrasts with increasing rates of overall LA, largely due to a rise in minor LA rates, in RI Saskatchewanians. If the rates of change continue on current trajectories, this disparity will continue to amplify. Further, regardless of population type, higher rates of LA were observed in males than females and individual aged 50+ years. These results underscore the need for more targeted intervention in these groups. Further investigation into contributing factors, including potential barriers to timely peripheral arterial disease care and diabetic foot care should be prioritized to reduce LA, especially in the RI population.
